# Discrepancy between central nervous system involvement at diagnosis and relapse burden in childhood acute lymphoblastic leukemia: a five-year cohort study in a middle-income setting

**DOI:** 10.3389/fonc.2026.1813121

**Published:** 2026-05-29

**Authors:** Carmen Gabriela Torres-Alarcón, Chantal Sandoval-López

**Affiliations:** 1 Department of Blood Bank, Hospital Central Militar, México City, Mexico; 2 Department of Pediatrics, Hospital General de Naucalpan, Naucalpan de Juárez, Estado de México, Mexico

**Keywords:** acute lymphoblastic leukemia, central nervous system, childhood acute leukemia, CNS involvement, CNS relapse, middle-income setting

## Abstract

**Background:**

Central nervous system (CNS) involvement is a critical prognostic factor in childhood acute lymphoblastic leukemia (ALL). Conventional cytology remains the diagnostic standard, but its limited sensitivity may lead to underdetection of CNS disease. This study evaluated the incidence and prognostic impact of CNS involvement at diagnosis and relapse in a Mexican pediatric cohort, focusing on the relationship between baseline CNS status and subsequent relapse.

**Methods:**

We conducted a retrospective cohort study of 73 children (<15 years) diagnosed with ALL at a national tertiary referral center between 2019 and 2020, with a five-year follow-up. CNS involvement was defined by cerebrospinal fluid cytology and classified as CNS-1, CNS-2, and CNS-3 according to international criteria. Incidence at diagnosis, CNS relapse during follow-up, and cumulative CNS involvement were estimated. CNS relapse was analyzed among patients achieving complete remission (n =57), excluding early deaths, to define the at-risk population. Survival was analyzed using Kaplan–Meier estimates and the log-rank test.

**Results:**

At diagnosis, CNS involvement occurred in 2.9% of evaluable patients (n =2/69; 95% CI: 0.8–9.5%), while 33.3% of patients were classified as CNS-2. During follow-up, CNS relapse occurred in 17.5% (n =10/57; 95% CI: 9.4–28.9%), with a cumulative proportion of 16.4% (n =12/73; 95% CI: 9.3–26.2%). Half of CNS relapses occurred in patients initially classified as CNS-2. CNS-3 status at diagnosis was associated with significantly inferior overall survival (log-rank, p = 0.007).

**Conclusion:**

Our findings identify a marked discordance between baseline CNS detection and subsequent relapse burden. The observed relapse burden in a cohort with a high prevalence of CNS-2 at presentation raises the possibility that conventional criteria may underestimate clinically relevant CNS disease in some patients. Prospective studies incorporating more sensitive diagnostic approaches are warranted to clarify the clinical implications of low-level cerebrospinal fluid blast detection.

## 1 Introduction

Acute lymphoblastic leukemia (ALL) is the most prevalent childhood cancer and a leading cause of cancer-related mortality. Historically, central nervous system (CNS) involvement contributed substantially to poor outcomes, with mortality rates reaching up to 75% (1). Recognition of the CNS as a critical disease site and the introduction of intrathecal prophylaxis transformed prognosis, enabling event-free survival rates to exceed 80% (1–3). Despite these advances, CNS involvement remains a clinically relevant challenge because of its association with relapse and adverse outcomes (4).

The CNS is the most frequently involved extramedullary site in ALL, both at diagnosis and relapse, and its involvement is associated with higher mortality, making it an unfavorable prognostic factor. Approximately 30 to 60% of extramedullary relapses in acute leukemia involve the CNS (2, 5). The reported incidence of CNS involvement at diagnosis ranges from 2% to 15% (6). Additional heterogeneity has been described within ALL, with precursor B-cell and T-cell subtypes showing distinct patterns of CNS involvement. These observations underscore the clinical relevance of CNS disease across acute leukemia subtypes and highlight the importance of accurately characterizing its incidence and behavior. Moreover, recent reviews and cohort studies suggest that clinically relevant CNS involvement may be underestimated at presentation, as subclinical disease may evade conventional diagnostic approaches, particularly when assessment relies exclusively on cytological examination (7, 8).

Cerebrospinal fluid cytology remains the gold standard for diagnosing CNS involvement. However, its reported sensitivity—approximately 50%—limits accurate case identification and may lead to underestimation of the true incidence of CNS disease. Consequently, irrespective of CNS status at diagnosis, all patients receive CNS-directed prophylactic treatment (2, 9). Complementary methods, such as flow cytometry, have enabled the identification of cases that would not have been detected by conventional cytology (6, 10). Nevertheless, their implementation is not yet routine in many medical centers, particularly in resource-limited settings.

In Mexico, epidemiological data regarding CNS involvement in childhood ALL—at diagnosis, relapse, and during long-term follow-up—remain sparse, limiting comparisons with international epidemiological series. This gap limits a comprehensive assessment of the impact of CNS involvement on clinical outcomes and timely identification of patients at risk. The objective of this study was to estimate the incidence of CNS involvement at diagnosis, relapse, and throughout follow-up in a pediatric cohort with ALL treated at a national referral center. Characterizing these patterns may inform and optimize CNS surveillance strategies in comparable resource-limited settings.

## 2 Materials and methods

### 2.1 Study design

An observational retrospective cohort study included patients aged <15 years with a *de novo* diagnosis of ALL treated at a national tertiary referral center between 1 January 2019 and 31 December 2020. All consecutive patients during the study period were included. A minimum dataset was required: complete blood count, immunophenotyping, baseline CNS evaluation, and clinical follow-up (at least until an event occurred or until the last documented contact). No additional exclusions were applied after eligibility screening. All patients were followed for five years after diagnosis.

### 2.2 Variables and operational definitions

Demographic variables (sex and age at diagnosis) and clinical variables were recorded, including the initial complete blood count (leukocyte count, hemoglobin, and platelet count). ALL subtypes were analyzed by immunological phenotype as B-ALL and T-ALL.

### 2.3 Classification of CNS status

The CNS status was defined by cytological examination of cerebrospinal fluid obtained by lumbar puncture and analyzed by cytocentrifugation (cytospin) with May–Grünwald–Giemsa staining according to the following criteria: CNS-1 was defined as non-traumatic puncture without leukemic blasts; CNS-2 as non-traumatic puncture with <5 leukocytes/µL and presence of leukemic blasts; and CNS-3 as non-traumatic puncture with ≥5 leukocytes/µL and presence of leukemic blasts. Non-traumatic lumbar puncture was defined as <10 erythrocytes/µL, and traumatic lumbar puncture (TLP) as ≥10 erythrocytes/µL. CNS involvement was considered positive only in cases classified as CNS-3 in the cytological report. Only cases confirmed by cytology were recorded as positive for CNS involvement.

### 2.4 Risk stratification and treatment

In patients with ALL, treatment was performed according to institutional protocols based on the BFM-95 (Berlin-Frankfurt-Münster) regimen. Risk stratification was performed prior to treatment based on age, initial white blood cell count, and leukemia phenotype (B/T), classifying patients as standard-risk of relapse (SR) or high-risk of relapse (HR). SR was defined as age ≥1 and <10 years and white blood cell count <50 × 10^9^/L, and HR as age ≥10 years and/or white blood cell count ≥50 × 10^9^/L.

CNS-directed therapy was administered according to risk group. Patients with SR received 11 doses of intrathecal methotrexate (IT-MTX), while patients with HR received 5 doses of IT-MTX and 6 doses of triple intrathecal therapy with doses adjusted for age and body surface area. Cranial irradiation was not used prophylactically; it was indicated only as treatment in patients with CNS involvement at diagnosis (CNS-3), who received therapeutic cranial radiotherapy (18 Gy) and two additional doses of IT-MTX.

### 2.5 Statistical analysis

Statistical analysis was performed using the Statistical Package for the Social Sciences (SPSS), version 31.0 (SPSS Inc., Chicago, IL, USA), with an institutional license. Quantitative variables were described using measures of central tendency and dispersion (mean and standard deviation), and categorical variables were expressed as frequencies and percentages. Age and initial white blood cell count were categorized using standard clinical cut-off points for risk stratification. The incidence of CNS involvement at diagnosis was calculated as the proportion of patients with CNS involvement identified at diagnosis, divided by the total number of patients included in the study. CNS relapse was calculated as the proportion of patients who developed CNS involvement during follow-up, regardless of their initial CNS status. To this end, an adjusted denominator was used for the relapse analysis; patients with TLP at diagnosis were included in the risk set upon achieving remission, while patients who suffered early death (defined as mortality occurring within the first 30 days post-diagnosis or during the induction phase) were excluded. This approach ensured that the numerator consisted only of relapse events in patients who successfully reached the period of risk. The cumulative proportion of CNS involvement was defined as the proportion of patients who developed CNS involvement, either at diagnosis or during follow-up, relative to the total number of patients included in the study. Exact (Clopper–Pearson) 95% confidence intervals were calculated for proportions. The association between clinical characteristics and the outcome (CNS involvement) at different measurement points (at diagnosis, at relapse, or during five-year follow-up) was evaluated using contingency tables and the chi-square (χ²) test. When expected cell counts were <5, Fisher’s exact test was used.

In addition, survival analysis was performed to evaluate the prognostic impact of CNS status at diagnosis on overall survival. Overall survival was defined as the time from diagnosis to death from any cause or, in the absence of an event, to the date of last recorded contact. Death was considered the event, and censoring time was defined as the interval (in years) from diagnosis to the event or the end of the observation period. Survival curves were estimated using the Kaplan–Meier method. For analyses stratified by CNS status at diagnosis, only patients with available non-traumatic lumbar puncture and definitive CNS classification (n=69) were included. Differences between groups (CNS-1, CNS-2, and CNS-3) were compared using the log-rank (Mantel–Cox) test. Follow-up time was expressed in years. In all analyses, a p-value < 0.05 was considered statistically significant. Given the limited number of events in certain subgroups, particularly the small number of patients classified as CNS-3 at diagnosis, multivariable Cox regression modeling was not performed.

### 2.6 Ethical considerations

This study was approved by the Research Ethics Committee of the Hospital Militar de Especialidades de la Mujer y Neonatología and was conducted in accordance with the principles of the Declaration of Helsinki. Owing to its retrospective nature, the requirement for informed consent was waived. Data were anonymized prior to analysis.

## 3 Results

### 3.1 Patient characteristics

A total of 73 patients were diagnosed with ALL during the study period. B-cell ALL was predominant, accounting for 89.0% (65), while T-ALL accounted for 11.0% (8). Slightly more than half of the patients were classified as high risk at diagnosis, 50.7% (37). Traumatic lumbar puncture with blasts was identified in four patients (5.5%) and analyzed separately. Baseline clinical and laboratory characteristics are presented in **Table 1**.

**Table 1:** Baseline characteristics of the study cohort.

Characteristic	n= 73	100%
Sex		
Male	49	67.1%
Female	24	32.9%
Age group (years)		
<1	3	4.1%
1 to 4	35	47.9%
5 to 9	21	28.8%
>10	14	19.2%
ALL (Immunophenotyping)		
B-ALL	65	89.0%
T-ALL	8	11.0%
Risk group		
Standard-risk	37	50.7%
High risk	36	49.3%
White blood cell count (×10^9/L)		
<10	41	56.2%
≥10 to <50	16	21.9%
≥50	16	21.9%
Hemoglobin (g/dL)		
<7	39	53.4%
7 to 11	29	39.7%
>11	5	6.8%
Platelets count (10^9/L)		
<20	29	39.5%
20 to 99	25	34.2%
>100	19	26.0%
CSF		
Non-TLP	69	94.5%
Positive TLP	4	5.5%
CNS status*		
CNS-1	44	63.8%
CNS-2	23	33.3%
CNS-3	2	2.9%

Data are presented as number (percentage).

CSF, cerebrospinal fluid; CNS, central nervous system; TLP, traumatic lumbar puncture with blasts.

*CNS status was assessed only in patients with a non-traumatic lumbar puncture (n= 69).

Risk group classification was based on institutional treatment protocols. Percentages may not total 100% due to rounding.

### 3.2 Central nervous system status at diagnosis

CNS status at diagnosis was evaluable in 69 patients with a non-traumatic lumbar puncture; among these patients, 63.8% (44) were classified as CNS-1, 33.3% (23) as CNS-2, and 2.9% (2) as CNS-3. **Table 2** summarizes the distribution characteristics according to CNS status at diagnosis among the 69 evaluable patients. Sex, age group, risk group classification, initial white blood cell count, hemoglobin levels, and platelet count at diagnosis were not significantly associated with CNS status. No statistically significant differences were observed across CNS categories for any of these variables (all p >0.05).

**Table 2:** Distribution of demographic and clinical variables by CNS status at diagnosis (non-traumatic LP only).

Variable	Category	Total		CNS-1		CNS-2		CNS-3		p value
		n=69	100%	n=44	63.8%	n=23	33.3%	n=2	2.9%	
Sex										
	Male	47	68.1%	32	72.7%	14	60.9%	1	50.0%	0.525
	Female	22	31.9%	12	27.3%	9	39.1%	1	50.0%	
Age group (years)										
	<1	3	4.3%	2	4.5%	1	4.3%	0	0.0%	0.775
	1–4	34	49.3%	24	54.5%	10	43.5%	0	0.0%	
	5–9	18	26.1%	10	22.7%	7	30.4%	1	50.0%	
	≥10	14	20.3%	8	18.2%	5	21.7%	1	50.0%	
ALL (Immunophenotyping)										
	B-ALL	61	88.4%	39	88.6%	20	87.0%	2	100.0%	0.856
	T-ALL	8	11.6%	5	11.4%	3	13.0%	0	0.0%	
Risk										
	Standard	33	47.8%	24	54.5%	9	39.1%	0	0.0%	0.190
	High	36	52.2%	20	45.5%	14	60.9%	2	100.0%	
White blood cell count (×10^9/L)										
	<10	39	56.5%	21	47.7%	16	69.6%	2	100.0%	0.240
	10–49	14	20.3%	12	27.3%	2	8.7%	0	0.0%	
	≥50	16	23.2%	11	25.0%	5	21.7%	0	0.0%	
Hemoglobin (g/dL)										
	<7	38	55.1%	25	56.8%	12	52.2%	1	50.0%	0.982
	7–11	26	37.7%	16	36.4%	9	39.1%	1	50.0%	
	>11	5	7.2%	3	6.8%	2	8.7%	0	0.0%	
Platelet count (×10^9/L)										
	<20	29	42.0%	22	50.0%	6	26.1%	1	50.0%	0.381
	20–99	24	34.8%	13	29.5%	10	43.5%	1	50.0%	
	≥100	16	23.2%	9	20.5%	7	30.4%	0	0.0%	

n=69 (Traumatic lumbar puncture with blasts was identified in four patients (5.5%) and analyzed separately).

CNS, central nervous system; WBC, white blood cell count. CNS status at diagnosis was evaluable in 69 patients with a non-traumatic lumbar puncture. Values are presented as n (% within CNS status at diagnosis).

Pearson’s chi-square test was used. In analyses where >20% of cells had expected counts <5, results should be interpreted with caution.

a; p values were obtained using Fisher’s exact test due to small, expected cell counts.

### 3.3 Central nervous system involvement at diagnosis and during follow-up

As shown in **Table 3**, CNS involvement at diagnosis and during follow-up was analyzed in the overall cohort of 73 patients. Four patients (5.5%) had a TLP with blasts; clinically, they were treated as CNS-3, but the analysis of CNS involvement at diagnosis included only patients with non-traumatic lumbar puncture (n =69). Overall, CNS involvement at diagnosis was 2.9% (n =2/69; 95% CI: 0.8-9.5%). The early death (defined as mortality within 30 days of diagnosis) occurred in 21.9% patients (n =16). As shown in **Table 4**, early mortality occurred more frequently in patients with CNS involvement at diagnosis (p = 0.009).

**Table 3:** Incidence of central nervous system involvement at diagnosis and during follow-up in acute lymphoblastic leukemia (n=73).

Leukemia (subtype)	n	TLP		Non-TLP		CNS involvement at diagnosis*		Patients without early death, overall (n)	CNS relapse		Cumulative CNS proportion**	
	Total	(n)	(%)	(n)	(%)	(n)	(%)		(n)	(%)	(n)	(%)
B-ALL	65	4	5.5%	61	95.5%	2	3.3%	52	8	15.4%	10	15.4%
T-ALL	8	0	0.0%	8	100%	0	0.0%	5	2	40.0%	2	25.0%
**ALL**	**73**	**4**	**5.5%**	**69**	**93.2%**	**2**	**2.9%**	**57**	**10**	**17.5%**	**12**	**16.4%**

ALL acute lymphoblastic leukemia; B-ALL, B-cell acute lymphoblastic leukemia; T-ALL, T-cell acute lymphoblastic leukemia; CNS, central nervous system; TLP, traumatic lumbar puncture.

*CNS involvement at diagnosis was evaluable in 69 patients with a non-traumatic lumbar puncture. Traumatic lumbar puncture with blasts was identified in four patients (5.5%) and was analyzed separately.

**Cumulative CNS proportion includes CNS involvement at diagnosis and during follow-up. Bold values correspond to the overall summary values for the complete acute lymphoblastic leukemia (ALL) cohort.

**Table 4:** Clinical characteristics associated with central nervous system involvement at diagnosis (n = 69).

Characteristic	All patients		CNS involvement at diagnosis				
			Yes		No		
	n	%	n	%	n	%	p value
	69	100.0	2	2.9	66	97.1	
Sex							
Male	47	68.1%	1	50.0%	46	68.7%	0.577
Female	22	31.9%	1	50.0%	21	31.3%	
Age group (years)							
<1	3	4.3%	0	0.0%	3	4.5%	0.484^a^
1 to 4	34	49.3%	0	0.0%	34	50.7%	
5 to 9	18	26.1%	1	50.0%	17	25.4%	
>10	14	20.3%	1	50.0%	13	19.4%	
ALL (Immunophenotyping)							
B-ALL	61	88.4%	2	100.0%	59	88.4%	0.603
T-ALL	8	11.6%	0	0.0%	8	11.6%	
Risk group							
Standard-risk	33	47.8%	0	0.0%	33	49.3%	0.169
High risk	36	52.2%	2	100.0%	34	50.7%	
White blood cell count (×10^9/L)							
<10	39	55.2%	2	100.0%	37	55.2%	0.453
≥10 to <50	14	20.6%	0	0.0%	14	20.9%	
≥50	16	23.9%	0	0.0%	16	23.9%	
Hemoglobin (g/dL)							
<7	38	55.1%	1	50.0%	37	55.2%	0.886
7 to 11	26	37.7%	1	50.0%	25	37.3%	
>11	5	7.2%	0	0.0%	5	7.5%	
Platelet count (×10^9/L)							
<20	29	42.0%	1	50.0%	28	41.8%	0.724
20 to 99	24	34.8%	1	50.0%	23	34.3%	
>100	16	23.2%	0	0.0%	16	23.9%	
Early death							
Yes	16	23.2%	2	100.0%	14	20.9%	0.009
No	53	76.8%	0	0.0%	53	79.1%	

^a^Fisher’s exact test (two-sided).

CNS, central nervous system; B-ALL, acute lymphoblastic leukemia.

For relapse proportions, early deaths were excluded because they did not reach the risk period. For CNS relapse analysis, the denominator was based on the population at risk, including all patients undergoing treatment regardless of initial CNS status; thus, patients with a traumatic lumbar puncture at diagnosis were included. Early deaths (n =16) were excluded from this specific analysis, as these patients died during induction and did not reach the relapse risk period. This exclusion applied to all such cases, including those with initial CNS-3 involvement (n =57).

During follow-up, CNS relapse occurred in 17.5% (10/57; 95% CI, 9.4–28.9), with variation across leukemia subtypes. Patients with T-ALL had a higher proportion of CNS relapse than those with B-ALL (2/5, 40.0% vs 8/52, 15.4%) (**Table 5**). When subgroup-specific proportions were examined, clinically relevant trends were observed. Patients aged 1–4 years had a lower proportion of CNS relapse (3/28, 10.7%) than those aged 5–9 years (5/19, 26.3%) or ≥10 years (2/8, 25.0%). CNS relapse was more frequent among patients classified as CNS-2 at diagnosis (5/19, 26.3%) than those classified as CNS-1 at diagnosis (4/34, 11.8%). Although these differences were not statistically significant (all p > 0.05), these trends warrant further investigation.

**Table 5:** Clinical characteristics associated with central nervous system relapse. (n = 57).

Characteristic	All patients		CNS Relapse				
			Yes		No		
	n	%	n	%	n	%	p value
	57	100.0	10	17.5	47	82.5	
Sex							
Male	39	68.4%	8	80.0%	31	66.0%	0.478^a^
Female	18	31.6%	2	20.0%	16	34.0%	
Age group (years)							
<1	2	3.5%	0	0.0%	2	4.3%	0.449
1 to 4	28	49.1%	3	30.0%	25	53.2%	
5 to 9	19	33.3%	5	50.0%	14	29.8%	
>10	8	14.0%	2	20.0%	6	12.8%	
ALL (Immunophenotyping)							
B-ALL	52	91.2%	8	80.0%	44	93.6%	0.208^a^
T-ALL	5	8.8%	2	20.0%	3	6.4%	
Risk							
Standard-risk	33	57.9%	3	30.0%	30	63.8%	0.054^a^
High risk	24	42.1%	7	70.0%	17	36.8%	
White blood cell count (×10^9/L)							
<10	33	57.9%	6	60.0%	27	57.4%	0.973
≥10 to <50	13	22.8%	2	20.0%	11	23.4%	
≥50	11	19.3%	2	20.0%	9	19.1%	
Hemoglobin (g/dL)							
<7	30	52.6%	6	60.0%	23	51.1%	0.678
7 to 11	24	42.1%	4	40.0%	20	42.6%	
>11	3	5.3%	0	0.0%	3	5.3%	
Platelet count (×10^9/L)							
<20	20	35.1%	2	20.0%	18	38.3%	0.143
20 to 99	19	33.3%	6	60.0%	13	27.7%	
>100	18	31.6%	2	20.0%	16	34.0%	
CNS- Status at diagnosis *							
CNS-1	34	64.2%	4	44.4%	30	68.2%	0.165^a^
CNS-2	19	35.8%	5	55.6%	14	31.8%	

*Analysis restricted to patients without CNS involvement at diagnosis who survived induction therapy (n =57).

CNS, central nervous system; ALL, acute lymphoblastic leukemia.

^a^Fisher’s exact test (two-sided)

**Figure 1:**
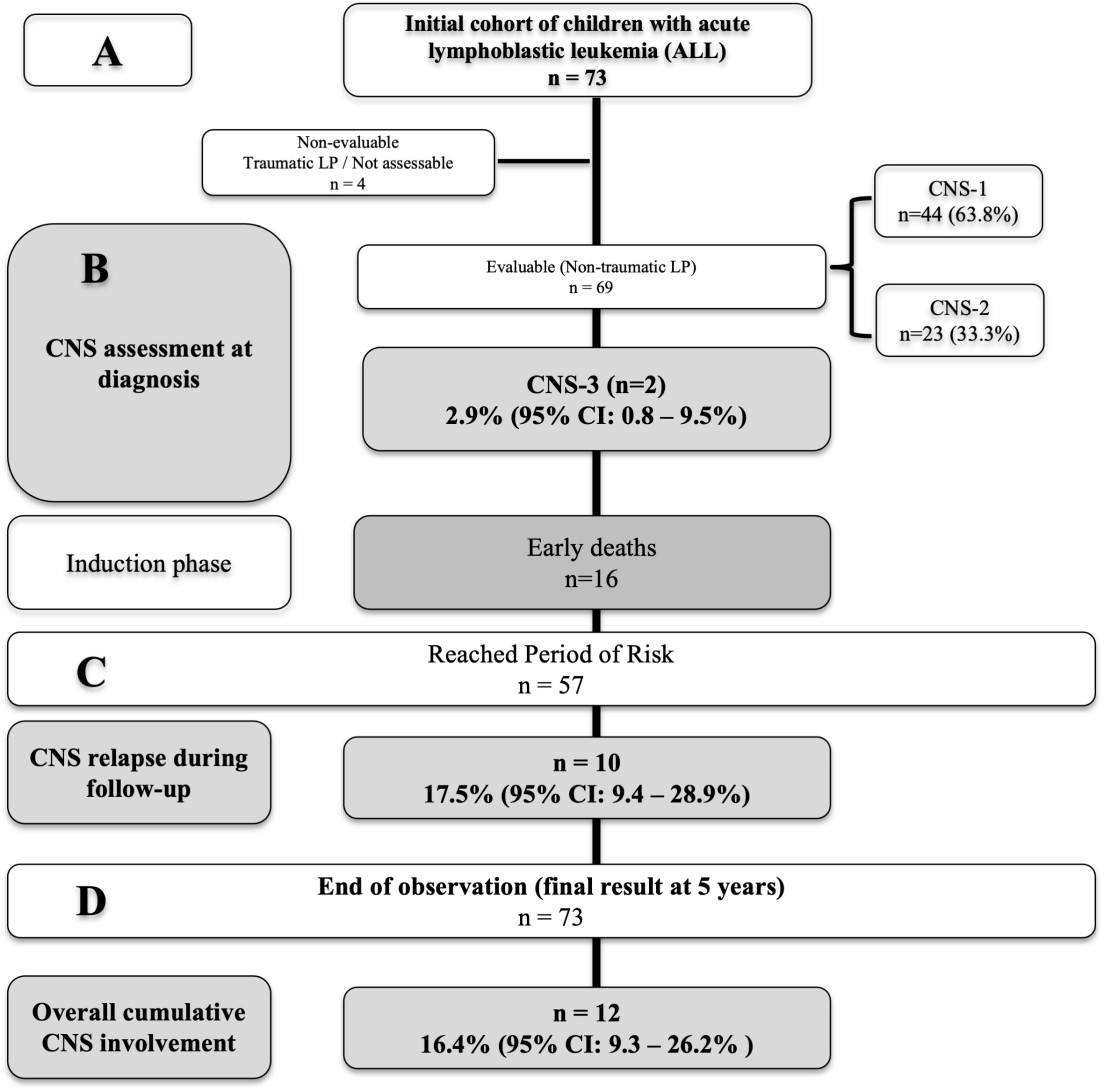
Longitudinal flow diagram of CNS involvement in childhood acute lymphoblastic leukemia. The diagram illustrates cohort dynamics from the initial diagnosis (2019–2020) to the conclusion of the 5-year study. **(A)** Initial cohort: Patients were diagnosed with acute leukemia during the study period (n=73). **(B)** CNS assessment at diagnosis: Detection and classification of CNS status among evaluable patients (n=69). **(C)** Follow-up Phase: Analysis of CNS relapse within the risk set (n=57) excluding 16 early deaths during induction. **(D)** End of Observation: Final cumulative incidence integrating all CNS events. The "n" values denote the population at risk each time point, and the "12/73" ratios indicate the number of cases detected relative to the corresponding risk set.

The cumulative proportion of CNS involvement—defined as CNS disease at diagnosis or during follow-up—was 16.4% (12/73; 95% CI, 9.3–26.2) (**Figure 1**). Similar subgroup-specific trends were observed. Higher proportions of CNS involvement were observed among patients aged 5–9 years (6/21, 28.6%) and ≥10 years (3/14, 21.4%) compared with those aged 1–4 years (3/35, 8.6%), and among T-ALL (2/8, 25.0% vs 10/65, 15.4% in B-ALL), although the difference was not statistically significant. Notably, patients with CNS-2 status at diagnosis had a higher proportion of CNS involvement during follow-up than those classified as CNS-1 (21.7% vs 9.1%) (**Table 6**). However, these findings should be interpreted with caution, given the limited number of CNS-3 cases and the potential influence of early mortality and loss to follow-up.

**Table 6:** Clinical and laboratory characteristics associated with cumulative CNS involvement during follow-up (n = 73).

Characteristic	All patients		CNS involvement during follow-up				
			Yes		No		
	n	%	n	%	n	%	p value
	73	100.0%	12	16.4%	61	83.6%	
Sex							
Male	49	67.1%	9	75.0%	40	65.6%	0.525
Female	24	32.9%	3	17.2%	21	34.4%	
Age group (years)							
<1	3	4.1%	0	0.0%	3	4.9%	0.197
1 to 4	35	47.9%	3	25.0%	32	52.5%	
5 to 9	21	28.8%	6	50.0%	15	24.6%	
>10	14	19.2%	3	25.0%	11	18.0%	
ALL (Immunophenotyping)							
B-ALL	65	89.0%	10	83.3%	55	90.2%	0.391
T-ALL	8	11.0%	2	16.7%	6	9.8%	
Risk group							
Standard risk	37	50.7%	3	25.0%	34	55.7%	0.050^a^
High risk	36	49.3%	9	75.0%	27	44.3%	
White blood cell count (×10^9/L)							
<10	41	56.2%	8	66.7%	33	56.2%	0.725
≥10 to <50	16	21.9%	2	16.7%	14	21.9%	
≥50	16	21.9%	2	6.7%	14	21.9%	
Hemoglobin (g/dL)							
<7	39	53.4%	7	58.3%	32	52.5%	0.588
7 to 11	29	39.7%	5	41.7%	24	39.3%	
>11	5	6.8%	0	0.0%	5	8.2%	
Platelet count (×10^9/L)							
<20	29	39.7%	3	25.0%	26	42.6%	0.157
20 to 99	25	34.2%	7	58.3%	18	29.5%	
>100	19	26.0%	2	16.7%	17	27.9%	
CNS status*							
CNS-1	44	63.8%	4	36.4%	40	42.6%	0.002
CNS-2	23	33.3%	5	45.5%	18	29.5%	
CNS-3	2	2.9%	2	18.2%	0	0.0%	

Analysis reflects cumulative proportion of CNS infiltration during follow-up, including all patients who developed CNS involvement at any time.

*CNS status was assessed only in patients with a non-traumatic lumbar puncture.

CNS, central nervous system; ALL, acute lymphoblastic leukemia.

^a^ Fisher’s exact test.

### 3.4 Overall survival according to CNS status at diagnosis

**Figure 2:**
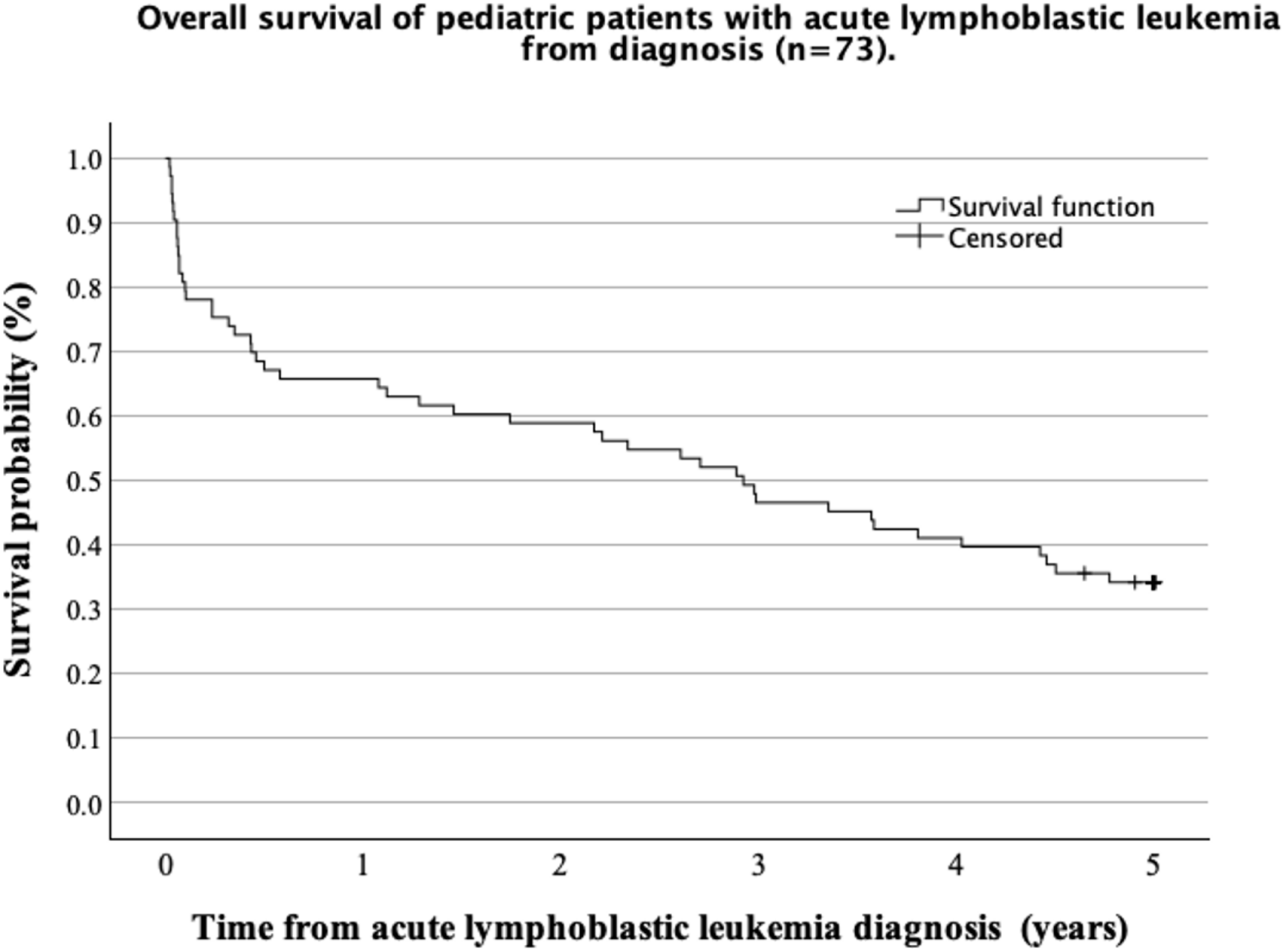
Overall survival of pediatric patients with acute lymphoblastic leukemia from diagnosis n=73. Kaplan–Meier estimates of overall survival from diagnosis over a 5-year follow-up period (n=73). Tick marks indicate censored observations.

Overall survival was analyzed using the Kaplan–Meier method in the entire cohort (n =73). During the five-year follow-up period, 48 deaths were recorded, while 25 patients (34.2%) were censored. The median overall survival for the entire cohort was 2.9 years (95% CI: 1.7–4.2) (**Figure 2**), and Kaplan–Meier analysis showed an estimated five-year overall survival of 34.2%.

**Figure 3:**
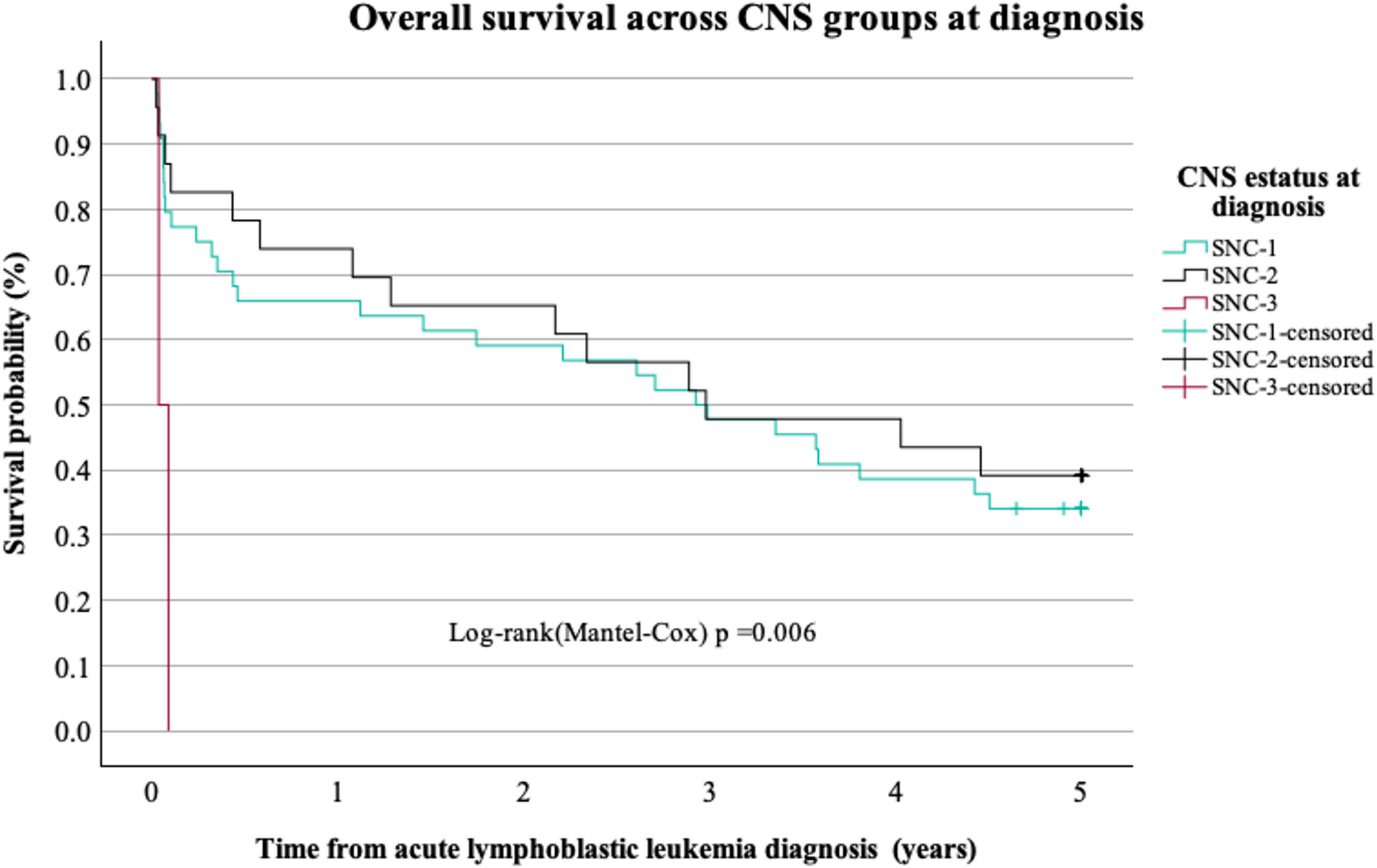
Kaplan–Meier estimates of overall survival stratified by central nervous system (CNS) status at diagnosis n=69. Analysis restricted to patients with non-traumatic lumbar puncture (n=69). Kaplan–Meier estimates of overall survival stratified by central nervous system (CNS) status at diagnosis (CNS-1, CNS-2, and CNS-3). Overall survival differed significantly according to CNS status (log-rank p < 0.006). Tick marks indicate censored observations. Should be interpreted with caution.

When survival was stratified by CNS status at diagnosis, only patients with non-traumatic lumbar puncture and a definitive CNS classification (n = 69) were included. Significant differences in overall survival were observed across CNS categories (log-rank, p < 0.006) (**Figure 3**). Patients classified as CNS-3 at diagnosis showed inferior survival compared with the CNS-1 and CNS-2 groups, with a median overall survival of 0.062 years. In contrast, median overall survival was 2.69 years (95% CI: 2.07–3.30) for CNS-1 patients and 2.94 years (95% CI: 2.11–3.78) for CNS-2 patients. These findings suggest that CNS involvement at diagnosis, particularly CNS-3 status, was associated with poorer overall survival.

## 4 Discussion

We estimated the incidence and clinical impact of CNS involvement in childhood ALL at diagnosis and over a five-year follow-up. At diagnosis, CNS involvement was low, but a much higher burden of CNS disease emerged during follow-up, with important prognostic implications. The observed discrepancy between the low frequency of CNS involvement at diagnosis and the higher burden of CNS relapse during follow-up suggests that, among other factors, limitations of conventional CNS assessment at presentation may contribute to this pattern. In this real-world cohort from a middle-income setting, this discordance may be clinically meaningful, raising the possibility that patients initially classified as CNS-negative or CNS-2 may harbor occult CNS disease undetected by conventional cytology. We cannot determine whether this observation results from a lack of treatment intensification in this subgroup, unmeasured biological risk factors (e.g., specific cytogenetics), or a combination of these factors.

The initial incidence of CNS-3 involvement was 2.9%, aligning with reports from large international consortia using conventional cytology (11–13). However, this diagnostic incidence alone does not capture the longitudinal burden of CNS disease, which becomes more evident when relapse events are included in a cumulative analysis. Despite the single-center design, the five-year follow-up provides a meaningful perspective on the longitudinal behavior of CNS disease. In contrast, CNS at relapse (17.5%) was higher than reported in international series (11, 14). This increase may be explained by lower loss to follow-up, a characteristic of a national referral center, and by less favorable clinical and biological factors, such as a high proportion of high-risk patients (49.3%) and elevated early mortality, phenomena previously described in the Mexican population (15). In the AIEOP-BFM ALL 2000 (11) cohort, more than 67% of patients were in the standard-risk group. CNS involvement at relapse was more frequent in T-ALL, raising the possibility of underdetection at diagnosis, since no patient with this subtype had CNS involvement at diagnosis. While our CNS involvement at diagnosis matches that of cytology-based cooperative series, the proportion of CNS relapse during follow-up is comparatively high, which may reflect the combined effects of baseline under-detection and context-specific clinical vulnerabilities. This observation is consistent with subgroup-specific trends in our cohort, including higher proportions of CNS involvement in older age groups and in T-ALL (**Tables 5**, **6**), although these differences were not statistically significant. This is also consistent with Martínez-Laperche et al. (16), who reported that CNS involvement at diagnosis identified only by flow cytometry predominantly corresponded to T-ALL. The high proportion of relapse may reflect a combination of adverse clinical profiles and potential baseline under-detection, especially in aggressive subtypes such as T-ALL. Although infants (<1 year) are known to have a higher propensity for CNS involvement, this subgroup represented a small proportion of our cohort (3/73) and was not associated with CNS status or CNS relapse, making it unlikely that age distribution significantly influenced our findings.

The cumulative proportion of CNS involvement was 16.4%, within the range reported in studies based only on cytology (14). Although cytology is accessible and widely used, its low sensitivity increases the risk of missing CNS involvement, which may contribute to subsequent relapse. Regarding the timing of recurrence among these 10 cases, 80% (n = 8) occurred after the completion of all chemotherapy cycles, while 20% (n = 2) occurred during the maintenance phase. The pattern observed (low incidence at diagnosis and a marked increase at relapse) may be explained by two complementary mechanisms. First, the true incidence of CNS involvement at diagnosis may be underestimated when cases that do not receive therapeutic intensification are missed. Second, some cases not detected at baseline could later manifest as CNS relapse, contributing to a higher incidence during follow-up or even early death before documentation. These mechanisms may be especially relevant in resource-constrained health systems, where diagnostic sensitivity, treatment intensity, and supportive care capacity collectively influence CNS disease control. These findings suggest that the cumulative proportion may be influenced by limitations of the diagnostic method and by the clinical complexity of subtle presentations or those coexisting with other neurological or infectious conditions (17). These limitations have been documented in large-scale diagnostic performance studies. Tabatabai et al. (18) reported difficulties in analyzing cerebrospinal fluid samples, with common incorrect diagnoses in inflammatory and hematopoietic malignancy categories, highlighting the potential for misclassification when cytology is the only diagnostic tool. Diagnostic underdetection is not the only possible explanation. The observed relapse burden may also be influenced by disease biology (e.g., T-lineage propensity), treatment-related variables, early mortality competing with relapse documentation, and health-system constraints that affect the timely delivery of protocol-intensity therapy and supportive care. The marked increase from the low initial detection rate (2.9%) to the high cumulative burden (16.4%) could reflect, at least in part, limitations of conventional cytological detection, although alternative explanations, such as disease biology and treatment-related factors, cannot be excluded.

Although cerebrospinal fluid cytology remains the gold standard for evaluating CNS involvement in acute leukemia, its diagnostic sensitivity is limited (16, 19). In our cohort, the incidence of CNS involvement at diagnosis was comparable to that reported in other international series based solely on cytology; however, studies using flow cytometry have shown discrepancies (7, 16, 19, 20). In the prospective study by De Haas et al. (19), cytology detected CNS involvement at diagnosis in 5% of patients, compared with 23% identified by flow cytometry. Similarly, Martínez-Laperche et al. (16) reported higher flow cytometry detection rates at relapse than cytology (27.8% vs 2.8%). Thastrup et al. (21) showed that 13% of relapses in children with ALL were identified exclusively by flow cytometry despite negative cytology. These findings are consistent with those reported by Levinsen et al. (20), who demonstrated that a proportion of CNS involvement cases occur without symptoms and that cytology may underestimate the actual tumor burden. Taken together, these findings suggest that cerebrospinal fluid cytology alone may underestimate the true burden of CNS disease, particularly in clinically silent or subclinical cases that may subsequently manifest as relapse. Although these studies originate primarily from high-income cooperative groups, their findings provide biological plausibility to the discrepancy observed in our cohort. This limitation may be explained by several pre-analytical and analytical factors affecting cerebrospinal fluid cytology. Pre-analytically, cerebrospinal fluid samples are highly susceptible to rapid cellular degradation; delays between collection and processing can compromise cellular integrity and increase the risk of false-negative results (18, 22). Analytically, morphological assessment is inherently subjective and operator-dependent, and distinguishing leukemic blasts from activated or reactive T-lymphocytes may be challenging, particularly in low-cellularity samples (22). These factors may contribute to underdetection and potential misclassification of CNS status (18, 19). Adjunctive techniques such as flow cytometry may improve diagnostic accuracy by enabling more precise characterization of cellular populations (15). The incorporation of more sensitive methods, such as flow cytometry in conjunction with cytology, may improve diagnostic precision and contribute to more refined risk stratification.

Beyond detection, CNS status at diagnosis has important prognostic implications. Schultz et al. (23) reported that patients classified as CNS-2 have outcomes similar to CNS-3 patients, partly because they do not receive the CNS-directed therapeutic intensification administered to patients in the CNS-3 category. These observations were derived from earlier treatment eras; subsequent cooperative group trials addressed this limitation by modifying therapeutic regimens, including increased CNS-directed therapy during induction for CNS-2 patients, which has been associated with improved CNS control and reduced relapse risk (24). In our cohort, survival differences were observed across CNS categories, likely influenced by early mortality in the two patients classified as CNS-3 at diagnosis. However, these findings should be interpreted with caution, as the very small number of patients classified as CNS-3 at diagnosis (n = 2) limits the robustness and stability of survival comparisons across CNS categories. In our cohort, the higher proportion of subsequent CNS relapse among CNS-2 patients compared with CNS-1, although not statistically significant, may reflect a different risk profile rather than a benign cytological finding. Nevertheless, given the limited sample size and observational design, these observations should be interpreted cautiously and do not support therapeutic modification. In our cohort, patients classified as CNS-2 did not receive additional CNS-directed therapeutic intensification beyond that assigned by risk stratification. Intensified intrathecal therapy may reduce relapse risk; however, its impact on overall survival remains uncertain and may be associated with increased toxicity in certain contexts (25, 26). Therefore, treatment decisions are multifactorial and not determined solely by CNS-2 status. In this real-world setting, variability in treatment delivery at the individual level cannot be excluded and may represent a source of unmeasured confounding in the interpretation of CNS relapse outcomes.

The relatively high proportion of patients classified as CNS-2 in our cohort (33.3%) appears higher than that reported in most published series and warrants careful interpretation. Several non-mutually exclusive explanations may account for this finding. First, variability in cytological interpretation and the inherent subjectivity of morphology-based assessment may influence classification thresholds across institutions. Second, pre-analytical factors may affect the detection and interpretation of low-level blast populations. Third, in the absence of more sensitive diagnostic techniques such as flow cytometry, borderline or low-level leukemic involvement may be classified as CNS-2 rather than being more precisely characterized. Notably, our baseline data did not show a clear concentration of CNS-2 cases within a distinct high-risk clinical subgroup, suggesting that methodological factors may have contributed to the observed frequency. Although CNS-2 has been hypothesized to represent occult or subclinical CNS disease, particularly in settings where more sensitive diagnostic techniques are not available, this interpretation remains uncertain in our cohort and may also reflect diagnostic and pre-analytical limitations. Therefore, CNS-2 findings in this context should be interpreted cautiously, considering both biological and methodological explanations. Future studies incorporating standardized diagnostic approaches and biological risk stratification will be necessary to clarify the clinical significance of CNS-2 classification.

Importantly, the five-year overall survival of 34.2% in our cohort falls within the wide range reported in low- and middle-income settings, where survival is highly heterogeneous (22–79%) (27, 28), with many centers reporting estimates around 40–65% (29). Consistent with this, a recent Mexican cohort reported a three-year overall survival of 74%, highlighting differences across centers and healthcare systems (30). These findings align with national reports from Mexico showing persistently high leukemia mortality and lower pediatric cancer survival than with other settings (31, 32). Within this context, our findings underscore the importance of optimizing CNS disease detection and control as part of broader efforts to improve outcomes in pediatric leukemia. Although our study was not designed to identify determinants of mortality, the high early mortality observed in our cohort was mainly attributable to infectious and hemorrhagic complications, likely related to severe cytopenias at presentation. Similar patterns have been reported in Mexican pediatric ALL cohorts, where infections and bleeding are leading causes of early mortality during the initial phases of treatment (15). Our findings also reinforce the clinical relevance of detecting any blasts in cerebrospinal fluid at baseline. Although CNS-2 does not meet the conventional threshold for ‘CNS involvement’, the higher proportion of subsequent CNS relapse in CNS-2 compared with CNS-1 may suggest a different risk profile; however, this interpretation remains uncertain and requires prospective validation.

Our study has several limitations that should be considered. The retrospective design and relatively small sample size, particularly the low number of CNS-3 cases, may limit statistical power and the robustness of subgroup analyses. In addition, CNS involvement was defined solely by conventional cerebrospinal fluid cytology, which has limited sensitivity and may lead to underestimation of disease at diagnosis and potential misclassification. The absence of flow cytometry may have contributed to the observed discrepancy between baseline CNS status and subsequent relapse burden. Furthermore, key biological variables, including cytogenetic and molecular abnormalities and minimal residual disease, were not consistently available, and treatment-related variables were not systematically captured, representing potential sources of unmeasured confounding. The use of proportion-based analyses may also limit the ability to fully account for competing risks such as early mortality. Therefore, these findings should be interpreted as exploratory and hypothesis-generating rather than as confirmatory. Despite these limitations, the study has notable strengths, including the homogeneity of the cohort, consistent diagnostic criteria, and five-year follow-up, which provide a valuable real-world perspective on CNS disease dynamics.

In conclusion, our study shows marked discordance between the low incidence of CNS involvement at diagnosis and the higher cumulative CNS relapse rate in pediatric ALL. While this pattern may reflect limitations of conventional CNS assessment, alternative explanations, including disease biology and treatment-related factors, cannot be excluded. Prospective studies incorporating more sensitive diagnostic approaches are needed to clarify the clinical implications of low-level cerebrospinal fluid blast detection. In settings where advanced diagnostic tools are not routinely available, careful longitudinal surveillance may be particularly important, although therapeutic implications remain uncertain and require validation in prospective studies incorporating biological and treatment-related variables.

## Data Availability

The data analyzed in this study is subject to the following licenses/restrictions: The datasets are not publicly available due to ethical and legal restrictions involving pediatric patient data. Data may be available from the corresponding author upon reasonable request and subject to institutional approval. Requests to access these datasets should be directed to dragabytorresalarcon@icloud.com.
